# *Chlorella* 11-Peptide Inhibits the Production of Macrophage-Induced Adhesion Molecules and Reduces Endothelin-1 Expression and Endothelial Permeability 

**DOI:** 10.3390/md11103861

**Published:** 2013-10-14

**Authors:** Mei Fen Shih, Lih Chi Chen, Jong Yuh Cherng

**Affiliations:** 1 Department of Pharmacy, Chia-Nan University of Pharmacy & Science, 60 Erh-Te Rd, Sec. 1, Tainan 71710, Taiwan; E-Mail: meifenshih@mail.chna.edu.tw; 2 Department of Pharmacy, Taipei City Hospital, Taipei 10341, Taiwan; E-Mail: lcchen@health.gov.tw; 3 Food and Drug Division, Department of Health, Taipei City Government, Taipei 11008, Taiwan; 4 Department of Biochemistry and Chemistry, National Chung Cheng University, 168 University Rd, Chia-Yi 62102, Taiwan

**Keywords:** endothelium, E-selectin, ICAM-1, VCAM-1, endothelin-1, intercellular permeability

## Abstract

The inflammation process in large vessels involves the up-regulation of vascular adhesion molecules such as endothelial cell selectin (E-selectin), intercellular cell adhesion molecule-1 (ICAM-1) and vascular cell adhesion molecule-1 (VCAM-1) which are also known as the markers of atherosclerosis. We have reported that *Chlorella* 11-peptide exhibited effective anti-inflammatory effects. This peptide with an amino sequence Val-Glu-Cys-Tyr-Gly-Pro-Asn-Arg-Pro-Gln-Phe was further examined for its potential in preventing atherosclerosis in this study. In particular, the roles of *Chlorella* 11-peptide in lowering the production of vascular adhesion molecules, monocyte chemoattractant protein (MCP-1) and expression of endothelin-1 (ET-1) from endothelia (SVEC4-10 cells) were studied. The production of E-selectin, ICAM-1, VCAM-1 and MCP-1 in SVEC4-10 cells was measured with ELISA. The mRNA expression of ET-1 was analyzed by RT-PCR and agarose gel. Results showed that *Chlorella* 11-peptide significantly suppressed the levels of E-selectin, ICAM, VCAM, MCP-1 as well as ET-1 gene expression. The inhibition of ICAM-1 and VCAM-1 production by *Chlorella* 11-peptide was reversed in the presence of protein kinase A inhibitor (H89) which suggests that the cAMP pathway was involved in the inhibitory cause of the peptide. In addition, this peptide was shown to reduce the extent of increased intercellular permeability induced by combination of 50% of lipopolysaccharide (LPS)-activated RAW 264.7 cells medium and 50% normal SEVC cell culture medium (referred to as 50% RAW-conditioned medium). These data demonstrate that *Chlorella* 11-peptide is a promising biomolecule in preventing chronic inflammatory-related vascular diseases.

## 1. Introduction

Circulating adhesion molecules (CAMs) are proteins expressed by vascular endothelium that are believed to play a role in the initiation of the atherosclerotic process. There are several families of CAMs, including integrins, cadherins, selectins and immunoglobulin superfamily members [1]. However, the most important adhesion molecules involved in atherosclerosis appear to be intercellular cell adhesion molecule-1 (ICAM-1), endothelial cell selectin (E-selectin), and vascular cell adhesion molecule-1 (VCAM-1). An elevated level of ICAM-1 has shown to correlate to an increased risk of cardiovascular events [2]. E-selectin levels correlate with cardiovascular disease (CVD) risk [3] and have been used successfully to predict the severity of atherosclerosis in patients [4]. VCAM-1 is a specific marker for advanced atherosclerosis, since it is often expressed in atherosclerotic plaques [5]. A high level of VCAM-1 is usually associated with an increased risk of coronary events in persons with existing CVD [2]. Studies also indicate that reducing the circulating level of these adhesion molecules could lower the CVD risk [6]. Up-regulation of the adhesion molecules on endothelial cells is prominent after these cells are exposed to pro-inflammatory molecules such as tumor necrosis factor (TNF)-α, interleukin (IL)-8, and monocyte chemoattractant protein-1 (MCP-1) [7]. Initiated by MCP-1, the recruitment and activation of monocytes/macrophages are followed and contribute to the initiation and pathophysiology of ischemic heart disease [8,9]. In addition, MCP-1 is believed to be involved in the development of atherosclerosis [10], coronary artery disease [11], postischemic myocardial remodeling [12], and heart failure [13]. Research on anti-MCP-1 gene therapy has successfully gained the attenuation of atherosclerosis in ApoE-deficient mice [14], suggesting MCP-1 as a potential therapeutic target in atherosclerosis.

Furthermore, endothelin-1 (ET-1) secreted by vascular endothelial cells acts as a potent endogenous vasoconstrictor and has an important role in the etiology of atherosclerotic vascular disease [15]. When endothelia are under inflammation, their increased intercellular permeability would also permit cholesterol uptake within the vessel wall [15]. Therefore, reducing the elevated ET-1 expression and remaining endothelial intercellular permeability to normal has been demonstrated to be an effective approach to block the development of circulatory disorders, including hypertension and atherosclerosis [16]. 

Green algae are able to prevent hyperlipidemia induced by a high fat diet [17] and atherosclerosis in laboratory animals [18]. Additionally, *Chlorella* 11-peptide derived from the green algae demonstrates various biological effects. The components of the *Chlorella* 11-peptide are (Val-Glu-Cys-Tyr-Gly-Pro-Asn-Arg-Pro-Gln-Phe). It has recently been shown to decrease inducible nitric oxide synthase (iNOS) expression and Nuclear factor-kappa-B (NF-κB) activity and to possess antioxidant properties [19,20]. Since atherosclerosis is known to be associated with an elevated cholesterol and chronic inflammation [21], examination of the relations between adhesion molecules, MCP-1, ET-1, endothelium membrane integrity with *Chlorella* 11-peptide would help to explore their therapeutic potential on atherosclerosis. In this study, the supernatant of LPS-stimulated RAW264.7 culture was added to endothelial cells (SVEC4-10) for stimulation of these cells. This is because activation of macrophage produces pro-inflammatory cytokines required for the development of atherosclerosis [22]. After the stimuli to SVEC4-10, responses of endothelia with and without *Chlorella* 11-peptide were then evaluated.

## 2. Results and Discussion

### 2.1. Inhibitory Effects of *Chlorella* 11-Peptide on LPS-Induced MCP-1 Production in RAW264.7 Macrophages

**Figure 1:**
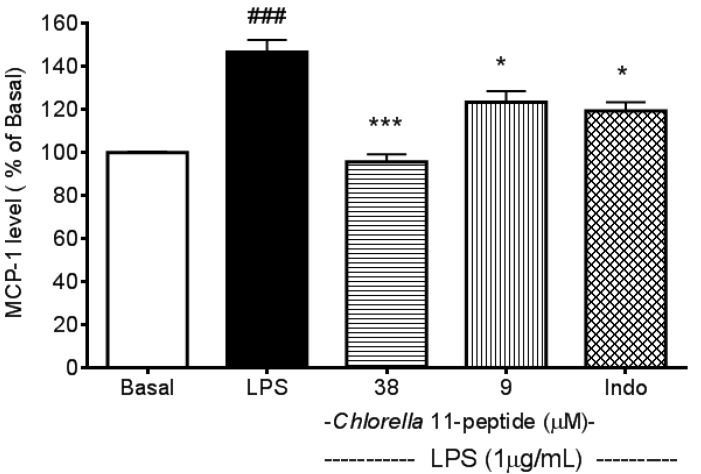
Effects of *Chlorella* 11-peptide on lipopolysaccharide (LPS)-induced monocyte chemoattractant protein (MCP-1) production. RAW264.7 cells (*n* = 8) were treated with LPS (1 µg/mL) with and without *Chlorella* 11-peptide (9 and 38 µM) or indomethacin (0.25 mM) for 6 h prior to MCP-1 concentration being measured. Statistics are shown for LPS-treated cells ### *p* < 0.005, compared to the basal; 38 µM of *Chlorella* 11-peptide *** *p* < 0.005 and 9 M of *Chlorella* 11-peptide and indomethacin * *p* < 0.05, compared to LPS-stimulated group.

The difference of MCP-1 production between LPS-treated and basal groups reached a maximum at 12 h after LPS stimulation [23]. Therefore, this time point was chosen for further study (*p* < 0.005, Figure 1). The LPS-induced MCP-1 production was significantly inhibited (*p* < 0.005 and *p* < 0.05) by *Chlorella* 11-peptide (38 µM and 9 µM, respectively). Indomethacin, a non-steroidal anti-inflammatory drug, also showed an inhibition on LPS-induced MCP-1 production. The inhibitory effect of indomethacin (0.25 mM) on MCP-1 production was found to be similar to that of *Chlorella* 11-peptide in a low dose (9 µM) but less potent in comparison to a high dose (38 µM) of *Chlorella* 11-peptide. 

Studies have shown that LPS induces production of proinflammatory cytokines (e.g., TNF-α, IL-1, IL-6) which contribute to vascular inflammation and atherosclerosis [19] and the occurrence of atherosclerosis is initiated by adhesion of monocytes to activated endothelial cells [21]. In the present study, we found that LPS significantly induced MCP-1 production in macrophages at 12 h (Figure 1). Therefore, the supernatant of LPS-stimulated macrophage cells cultured at 12 h was applied to induce adhesion molecules in SVEC4-10 endothelial cells and these induced molecules in relation to *Chlorella* 11-peptide can then be evaluated. 

### 2.2. Inhibitory Effects of *Chlorella* 11-Peptide on E-Selectin, ICAM-1 and VCAM-1 Production Induced by 50% RAW-Conditioned Medium

**Figure 2:**
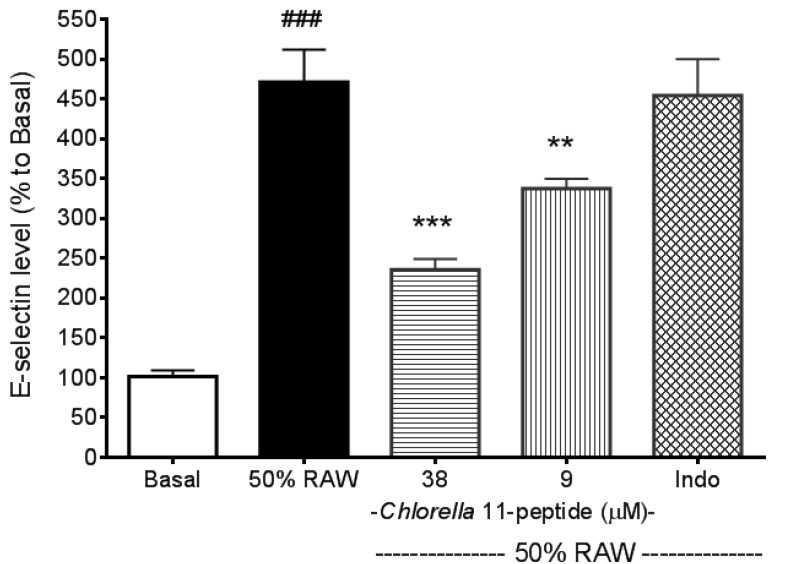
Effects of *Chlorella* 11-peptide on 50% RAW-conditioned medium-induced E-selectin production. SVEC4-10 endothelial cells (*n* = 8) were treated with 50% RAW-conditioned medium with and without *Chlorella* 11-peptide (9 and 38 µM) or indomethacin (0.25 mM) for 24 h prior to E-selectin concentration being measured. Statistics are shown for 50% RAW-conditioned medium-treated cells ### *p* < 0.005, compared to the basal; 38 µM of *Chlorella* 11-peptide *** *p* < 0.005 and 9 µM of *Chlorella* 11-peptide ** *p* < 0.01, compared to 50% RAW-conditioned medium-treated group.

**Figure 3:**
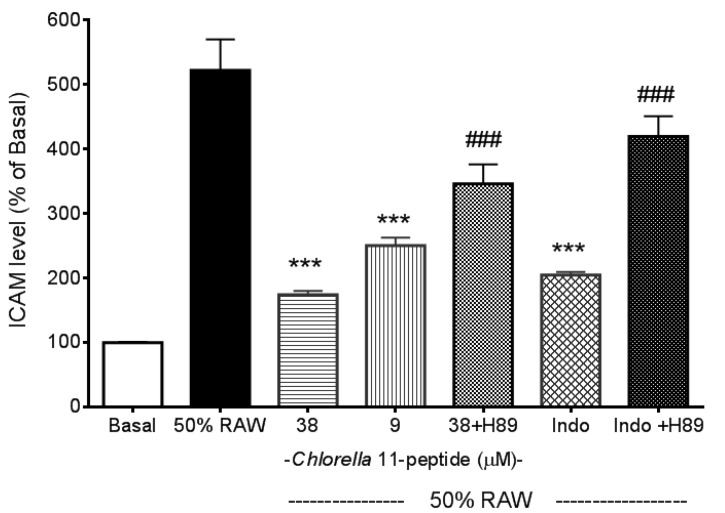
Effects of *Chlorella* 11-peptide on 50% RAW-conditioned medium-induced ICAM-1 production. SVEC4-10 endothelial cells (*n* = 8) were treated with 50% RAW-conditioned medium with and without *Chlorella* 11-peptide (9 and 38 µM) or indomethacin (0.25 mM) for 24 h prior to ICAM-1 concentration being measured. Statistics are shown for 9 µM and 38 µM of *Chlorella* 11-peptide, and indomethacin ### *p* < 0.005 compared to 50% RAW-conditioned medium-treated group; 38 µM of *Chlorella* 11-peptide+H89 and indomethacin+H89, *** *p* < 0.005, compared to 38 µM of *Chlorella* 11-peptide and indomethacin.

**Figure 4:**
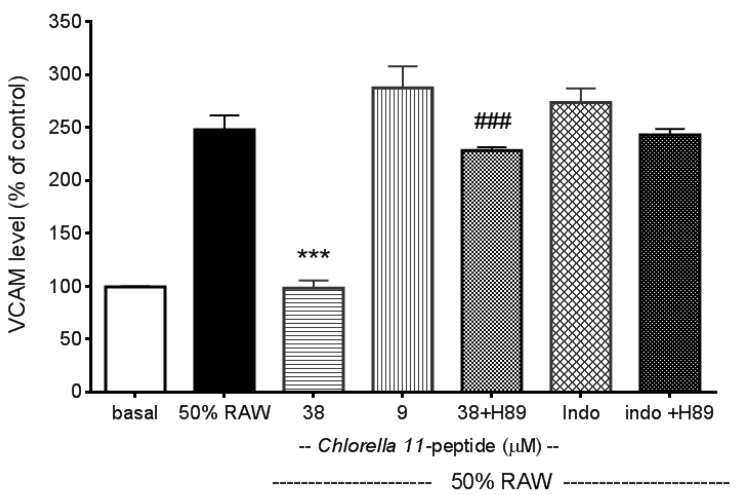
Effects of *Chlorella* 11-peptide on 50% RAW-conditioned medium-induced VCAM-1 production. SVEC4-10 endothelial cells (*n* = 8) were treated with 50% RAW-conditioned medium with and without *Chlorella* 11-peptide (9 and 38 µM) or indomethacin (0.25 mM) for 6 h prior to VCAM-1 concentration being measured. Statistics are shown for 38 µM of *Chlorella* 11-peptide, *** *p* < 0.005 compared to 50% RAW-conditioned medium-treated group; 38 µM of *Chlorella* 11-peptide+H89, ### *p* < 0.005, compared to 38 µM of *Chlorella* 11-peptide.

Recombined MCP-1 and other proinflammatory cytokines (recombined TNF-α and IL-6), as mentioned in the introduction of the manuscript, are capable of inducing adhesion molecules. These evidences were all well demonstrated by others’ work and have been tested in our preliminary experiments (data not shown). In addition, in our experiments we also found that *Chlorella* 11-peptide-treated RAW medium was not able to induce adhesion molecules production as a control (data not shown). E-selectin production in SVEC4-10 endothelial cells was significantly induced by 50% RAW conditioned medium (*p* < 0.005, Figure 2). Both the high (38 µM) and the low (9 µM) concentrations of *Chlorella* 11-peptide were able to significantly decrease the E-selectin production (*p* < 0.005 and *p* < 0.01, respectively). However, indomethacin did not affect the production of E-selectin induced by 50%RAW-conditioned medium. There was about a 5-fold increase in ICAM-1 production when SVEC4-10 endothelial cells were stimulated with 50%RAW-conditioned medium (Figure 3). The increased ICAM-1 production was significantly inhibited by *Chlorella* 11-peptide and indomethacin (*p* < 0.005, Figure 3). VCAM-1 production was also induced with 50% RAW-conditioned medium (Figure 4). Notably, neither the low concentration of *Chlorella* 11-peptide nor indomethacin exhibited any inhibition on VCAM-1 induction. However, the high concentration of *Chlorella* 11-peptide significantly suppressed the VCAM-1 production (*p* < 0.005). 

Normally E-selectin expressed by endothelial cells is low but cytokines, reactive oxygen species, and bacterial endotoxin can elicit its expression [24]. ICAM-1 is constitutively expressed on endothelial cells in most regional vascular beds, and its expression can be significantly increased with cytokines or bacterial endotoxin. In comparison with ICAM-1, VCAM-1 predominantly mediates the adhesion of lymphocytes and monocytes upon stimulation [25]. Amberger *et al*. reported a low VCAM-1 gene expression of human umbilical vein endothelial cells after TNF-α stimulation [26]. Importantly, all of these elevated adhesion molecules in positive relation to atherosclerosis were substantially reduced by the presence of *Chlorella* 11-peptide (38 µM). Indomethacin is a potent antiinflammatory agent. However, its inhibitory effects on proinflammatory cytokine-induced adhesion molecules (e.g., E-selectin, VCAM-1) were not as effective as shown in Figure 2 and Figure 4. Moreover, compared to indomethacin (Figure 4), *Chlorella* 11-peptide can effectively alleviate both the production of ICAM-1 and VCAM-1 which are responsible for leukocyte transmigration (ICAM-1) and leukocyte-endothelium signal transduction (VCAM-1) [27,28]. 

Cyclic AMP is a ubiquitous regulator of inflammatory and immune reactions. Mediation of cell cross talk through adhesion molecules (e.g., ICAM-1, E-selectin, VCAM-1) has been reported to be dependent on intracellular cAMP [29,30,31]. In this study, we used an inhibitor of protein kinase A, H89 [26], to study whether inhibitory effects of *Chlorella* 11-peptide on ICAM-1 and VCAM-1 production were mediated via the cAMP pathway. We showed that addition of H89 can compromise the inhibitory effects of *Chlorella* 11-pepetide on the induced ICAM-1 and VCAM-1 production (Figure 3 and Figure 4). This indicates that cAMP pathway was likely involved in the inhibitory actions of the peptide. The counteraction of H89 to indomethacin on the induced VCAM-1 production was not observed (Figure 4). The possible explanation is that Indomethacin requires much higher concentration (above 20 mM) to inhibit cAMP-dependent protein kinase activity [32], which is much higher than the concentration (0.25 mM) used in this study.

### 2.3. Inhibitory Effects of *Chlorella* 11-Peptide on Endothelin-1 Gene Expression

**Figure 5:**
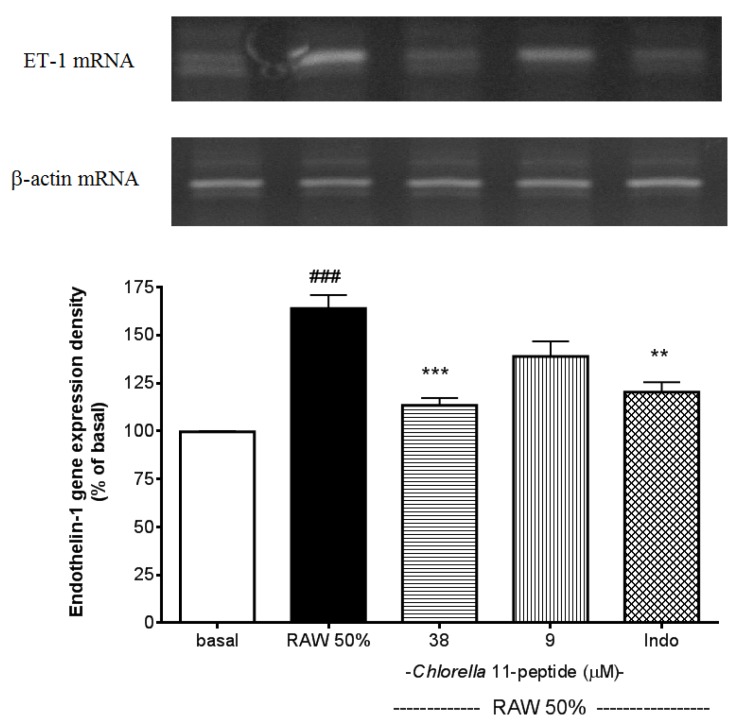
Effects of *Chlorella* 11-peptide on 50% RAW-conditioned medium-induced endothelin-1 mRNA expression. SVEC4-10 endothelial cells (*n* = 8) were treated with 50% RAW-conditioned medium with and without *Chlorella* 11-peptide (9 and 38 µM) or indomethacin (0.25 mM) for 24 h prior to total RNA extraction and PCR were performed. Statistics are shown for 50% RAW-conditioned medium-treated cells ### *p* < 0.005 compared to the basal; 38 µM of *Chlorella* 11-peptide *** *P* < 0.005 and indomethacin ** *p* < 0.01, compared to 50% RAW-conditioned medium-treated group.

The addition of 50% RAW-conditioned medium strongly induced the endothelin-1 (ET-1) mRNA expression in SVEC4-10 endothelial cells (*p* < 0.005, Figure 5). This induction of ET-1 gene expression can be significantly inhibited by the high dose of *Chlorella* 11-peptide (*p* < 0.005) and indomethacin (*p* < 0.01).

ET-1 mRNA expression was reported to be elevated in the artery with atherosclerotic lesion [16]. Antagonism of the ET-1 receptors was able to reduce the atherosclerotic lesion formation [33]. Thus, blocking ET-1 has been considered to be a strategy for prevention of atherosclerosis [15]. In this study, data showed that *Chlorella* 11-peptide is able to significantly suppress the ET-1 mRNA expression and holds a great potential in anti-atherosclerosis uses.

### 2.4. Inhibitory Effects of *Chlorella* 11-Peptide on Intercellular Permeability of Endothelia

**Figure 6:**
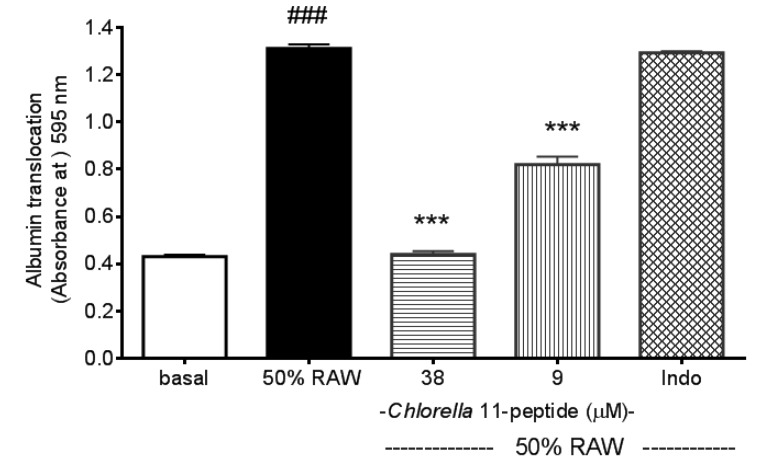
Effects of *Chlorella* 11-peptide on 50% RAW-conditioned medium-induced intercellular permeability. SVEC4-10 endothelial cells (*n* = 8) were treated with 50% RAW-conditioned medium with and without *Chlorella* 11-peptide (9 and 38 µM) or indomethacin (0.25 mM) for 24 h prior to E-selectin concentration being measured. Statistics are shown for 50% RAW-conditioned medium-treated cells ### *p* < 0.005, compared to the basal; 9 µM and 38 µM of *Chlorella* 11-peptide *** *p* < 0.005, compared to 50% RAW-conditioned medium-treated group.

After stimuli of 50% RAW-conditioned medium, the SVEC4-10 endothelial intercellular permeability showed a great increase (*p* < 0.005, Figure 6). Both the high and the low doses of *Chlorella* 11-peptide inhibited the increased intercellular permeability (*p* < 0.005). Moreover, this inhibition on the intercellular permeability was not observed in the indomethacin-treated group. 

Endothelial permeability is controlled in part by the dynamic opening and closing of endothelial cell-cell junctions which relates to the interactions between endothelial cells and the extracellular matrix [34,35]. Increased endothelial permeability to lipoproteins or immune cells is considered as an initiating step of atherosclerosis pathogenesis, and the accumulation of debris in the intima would result in atherosclerotic plaques [21]. Therefore, maintaining the endothelial permeability unaffected by proinflammatory cells would help to decrease the plaque formation [36]. Our permeability assay showed that *Chlorella* 11-peptide possesses a potent ability to inhibit the increased intercellular permeability of SVEC4-10 endothelia induced by 50% RAW-conditioned medium (Figure 6), which indicates the effectiveness of the peptide in preventing the development of atherosclerosis.

## 3. Experimental Section

### 3.1. Materials

Dulbecco’s modified eagles medium (DMEM), sodium pyruvate and non-essential amino acid were purchased from Gibco BRL (Taipei, Taiwan). Indomethacin (I17378), papain, pepsin, Bradford reagent (B6919), and H89 were purchased from Sigma (Taipei, Taiwan). Flavourzyme Type A and alcalase were purchased from Novo Nordisk (Taipei, Taiwan). A Total RNA Miniprep System (Viogene BioTek Corporation, Taipei, Taiwan) and Access RT-PCR (Promega, Madison, WI, USA) were used for RT-PCR. MCP-1, VCAM, ICAM and E-selectin ELISA assay kits were products from R&D systems (Taipei, Taiwan). All chemicals were dissolved in sterilized deionized H_2_O except for indomethacin in 95% aqueous ethanol and pepsin in cold 10 mM HCl (4 mg mL^−^^1^).

### 3.2. *Chlorella*-11 Peptide Preparation

*The Chlorella-11* peptide of *Chlorella pyrenoidosa* was prepared from algal protein waste essentially as described previously [19,20]. The algal protein waste was obtained from the insoluble material after hot water extraction of *Chlorella pyrenoidosa*. Then, the algal protein waste (10%, w/v) was digested with commercial proteases (pepsin, flavourzyme, alcalase, and papain) at the concentration of 0.2% (w/v) for 15 h at the designate pH and temperature for each enzyme reaction, using the reaction conditions provided by the manufacturer. At the end of the reaction, the digestion was heated in a boiling water bath for 10 min in order to inactivate the enzyme. The resulted non-soluble material was subsequently spray-dried and was extracted with methanol (1:10 w/v) for three times. After being concentrated, the MeOH-extracted fraction was then extracted by distilled water again prior to freeze-drying. 

The isolation and purification of *Chlorella* 11-peptide was as described previously with modification [19,20]. Briefly, ammonium sulfate was added to precipitate proteins and the precipitated proteins were removed by centrifugation (10,000× *g*, 30 min) and dissolved in a small volume of distilled water. The solution was fractionated using a Sephacryl S-100 high HR column (ϕ 2.6 × 70 cm) to detect peptides at OD 210 nm. The fraction was collected and subsequently loaded onto a Q-sepharose Fast Flow column (ϕ 2.6 × 40 cm), which was pre-equilibrated with a 20 mM Tris-HCl buffer solution (pH 7.8). The separation was performed with 1.0 M NaCl in the same buffer solution and fractions were collected, dialyzed in deionized water, and lyophilized. The peptide concentration was determined with Pierce micro BCA protein assay (Thermo Fisher Scientific Inc., Waltham, MA, USA). The molecular weight of peptides was determined by using a Superdex peptide HR 10/30 column at a flow rate of 0.5 mL/min. The standard curve was established with cytochrome c (MW 12327 Da), approtonin (MW 6500 Da), gastrin (MW 2098 Da) and Leu-Gly (MW 188 Da). 

The peptide fraction was further separated successively by a reversed-phase HPLC column, and a purified peptide was attained. The purity and identity of the final preparation was confirmed by mass spectrometric analysis on an Agilent 6510 Q-TOF mass spectrometer (>95%) and Edman degradation (see Supplementary Information). The sequence of *Chlorella* 11-peptide was determined as Val-Glu-Cys-Tyr-Gly-Pro-Asn-Arg-Pro-Gln-Phe, with a molecular mass of 1309 Da. In later parts of our work, we used the synthetic peptide provided by Kelowna International Scientific Inc. (Taiwan).

### 3.3. RAW264.7 Macrophage Culture

Macrophage RAW264.7 (ATCC number: TIB-71) cells were obtained from Bioresource Collection & Research Center (Taiwan) and cultured in DMEM supplemented with 10% Fetal Bovine Serum (Hyclone), 2 mM glutamine, 1% non-essential amino acid, 25 mM HEPES, 1 mM sodium pyruvate, 100 U/mL penicillin and 100 µg/mL streptomycin. Cells were cultured and maintained in 75T flasks (1 × 10^7^ cells/dish) at 37 °C in a humidified atmosphere containing 5% CO_2_.

### 3.4. MCP-1 Assay

The RAW264.7 cells were seeded at a density of 2 × 10^4^ cells/well in 96-well plates. Subsequently, the cells were incubated in the presence of LPS (1 µg/mL) with/without *Chlorella* 11-peptide (9 µM and 38 µM) for 12 hours prior to the culture medium was collected for MCP-1 measurement. This culture supernatant (without peptide treatment) was also taken to prepare “50% RAW-conditioned medium” (see the next section). The concentration range of *Chlorella* 11-peptide was chosen based on our previous finding [19]. Indomethacin (0.25 mM) was used as a positive control. MCP-1 levels in culture media were determined with commercial ELISA assay kits.

### 3.5. Adhesion Molecules—E-Selectin, ICAM-1 & VCAM-1 Level Measurements in SVEC4-10 Endothelial cells

SVEC4-10 cells (ATCC number: CRL-2181), which are well differentiated, responding like normal endothelial cells to some interleukins and to extracellular matrix signals for tube-like differentiation. SVEC4-10 was demonstrated to retain morphological and functional characteristics of normal EC [37]. SVEC4-10 endothelial cells were obtained from Bioresource Collection & Research Center (Taiwan) and cultured in Dulbecco’s Modified Eagle’s Medium (DMEM) supplemented with 10% Fetal Bovine Serum, 1 mM sodium pyruvate, 100 U/mL penicillin and 100 µg/mL streptomycin. Cells were maintained in 100 mm petri dishes at 37 °C in a humidified atmosphere containing 5% CO_2_. The SVEC4-10 cells were seeded at a density of 2 × 10^4^ cells/well in 96-well plates overnight prior to treatments. For stimuli, the culture medium of SVEC4-10 cells was changed to “50% RAW-conditioned medium” (made of equal volume of SVEC4-10 medium and the supernatant of LPS-stimulated RAW264.7 culture medium) with and without *Chlorella* 11-peptide. Indomethacin was dissolved in 95% ethanol and applied as a positive control and cells were incubated for further 24 h prior to E-selectin and ICAM-1 assays and 6 h prior to VCAM-1 assay. The concentration of these adhesion molecules were measured with commercial ELISA assay kits. 

### 3.6. Endothelin-1 mRNA Analysis by Reverse Transcription-Polymerase Chain Reaction (RT-PCR)

SVEC4-10 endothelial cells (2 × 10^5^ cells/mL) in a 100 mm dish were stimulated with 50% RAW-conditioned medium in the presence of *Chlorella* 11-peptide and incubated for 24 h. Total RNA was extracted by using a GENTRA RNA isolation kit (R-5000A, Minneapolis, MN, USA). The RNA was reverse transcribed using a first strand cDNA synthesis kit for RT-PCR (Access RT-PCR system, Promega, Madison, WI, USA). Semi-quantitative PCR was performed using primers for mouse ET-1 (forward, 5′-AAGCGCTGTTCCTGTTCTTCA-3′; reverse, 5′-CTTGATGCTATTGCTGATGG-3′) and housekeeping gene β-actin (forward, 5′-GTGGGCCGCTCAGGCCA-3′; reverse, 5′-CTCAGCTGTGGTGGTGAAGC-3′). Reaction products were examined by electrophoresis in 1% agarose gel and visualized with ethidium bromide. Densitometric analysis was performed using the Alpha Imager 2000 Documentation & Analysis System (Alpha Innotech Corporation, San Leandro, CA, USA).

### 3.7. BSA Transwell Permeability Assay

SVEC4-10 cells were seeded (1 × 10^5^ cells/insert) on gelatin-coated (1%) polystyrene filters (Costar Transwell, pore size = 0.4 mm, Corning Inc., Taiwan), allowed to grow to confluence on Transwell inserts and then replaced with FBS-free medium for additional 3 h [38]. An FBS-free medium containing 10 mg/mL BSA was placed into the upper compartment and an FBS-free medium without BSA was placed in the lower compartment of the Transwell. The transfer rate across the cell monolayer was assessed by measuring the increased amount of BSA in the lower compartment after 30 min. BSA was quantified using Bradford reagent.

### 3.8. Statistical Analysis

Data of results (*n* ≥ 8) from different experimental days were statistically analyzed for MCP-1, E-selectin, ICAM-1, VCAM-1, and intercellular permeability assays using Prism software (GraphPAD Inc., La Jolla, CA, USA). A two-tailed Student’s unpaired test was applied to compare the mean values of two populations of continuous data. The normal distribution and variance of each group was the same. Electrophoresis gel data were performed (*n* ≥ 3) and a representative example is shown in the results. 

## 4. Conclusions

The expression of various CAMs by activated endothelial cells is a rate-determining step in recruiting inflammatory cells and plays a pivotal role in the progress of CVD [6,24]. Our previous study clearly showed that peptides from *Chlorella* are a very effective inhibitor of TNF-α and IL-6 production in macrophages [19]. In this study, we further demonstrate that *Chlorella* 11-peptide could not only prevent LPS-induced MCP-1 production in RAW264.7 macrophages, but also effectively inhibit the production of adhesion molecules in endothelial cells. In addition, this peptide exhibited to alleviate ET-1 mRNA expression and maintain endothelial permeability unaffected under the influence of proinflammatory cytokines. By combining these results, *Chlorella* 11-peptide may become a potential biomolecule in ameliorating the development of atherosclerosis. 

## Supplementary Files

Supplementary File 1 Supplementary Materials (PDF, 27 KB)
