# Supplementary material for: Chlorella 11-Peptide Inhibits the Production of Macrophage-Induced Adhesion Molecules and Reduces Endothelin-1 Expression and Endothelial Permeability
Source: Mar Drugs. 2013 Oct 14;11(10):3861–74. doi: 10.3390/md11103861 (PMC3826139; doi:10.3390/md11103861)

Supplementary Materials

Figure S1. HPLC conditions for *Chlorella* 11-peptide are described. Column: 4.6 × 250 mm, Venusil XBP-C18. An elution system consisted with two solvents: (a) 0.1% trifluoroacetic acid in 100% acetonitrile and (b) 0.1% trifluoroacetic acid in 100% water. A mixture of 18% solvent (a) and 82% solvent (b) was applied to the column with a flow rate of 1.0 mL/min. The retention time of the peptide appeared at 12.6 min with absorption monitored at 220 nm.

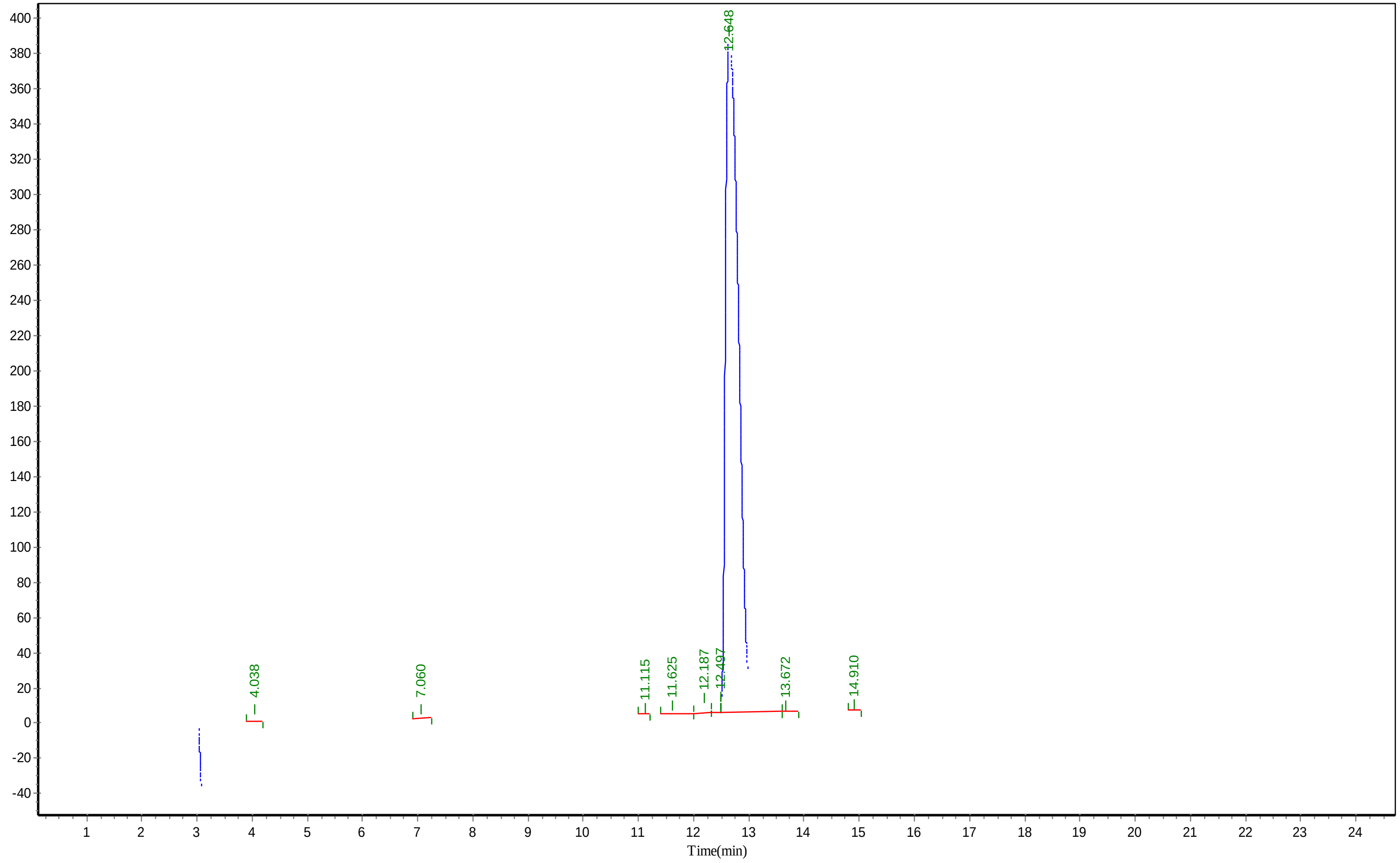

Figure S2. For the MS experiment using a Q-TOF mass spectrometer, typical source parameters were capillary voltages of 3 kV and cone voltages of 50 V, MS experiments were performed in the collision cell of the instrument and argon was used as a collision gas, a representative spectrum shows a signal of [*Chlorella*-11 peptide + H]$^+$ at m/z 1310 as depicted below.

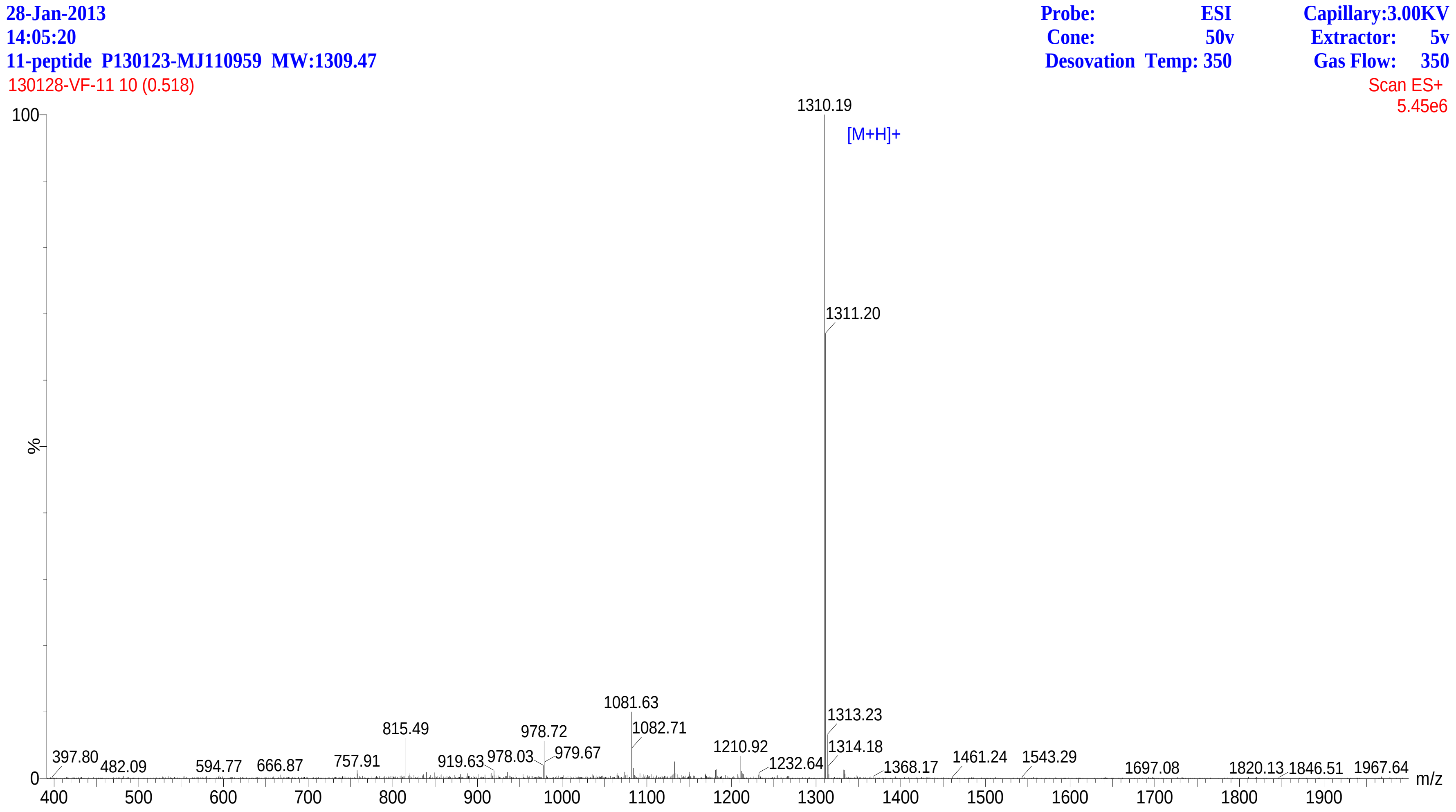

Supplement: Supplementary File 1 — Supplementary Materials (PDF, 27 KB) [file marinedrugs-11-03861-s001.pdf]
